# Advances in the Molecular Genetics of Catecholaminergic Polymorphic Ventricular Tachycardia

**DOI:** 10.3389/fphar.2021.718208

**Published:** 2021-08-16

**Authors:** Junxia Song, Yanhong Luo, Ying Jiang, Jianfeng He

**Affiliations:** ^1^ Departments of Cardiology, Ministry of Education Key Laboratory of Child Development and Disorders, National Clinical Research Center for Child Health and Disorders (Chongqing), China International Science and Technology Cooperation Base of Child Development and Critical Disorders, Children’s Hospital of Chongqing Medical University, Chongqing, China; ^2^ Endocrinology, Ministry of Education Key Laboratory of Child Development and Disorders, National Clinical Research Center for Child Health and Disorders (Chongqing), China International Science and Technology Cooperation Base of Child Development and Critical Disorders, Children’s Hospital of Chongqing Medical University, Chongqing, China

**Keywords:** catecholaminergic polymorphic ventricular tachycardia, genes, pathogenesis, treatments, diagnosis

## Abstract

Catecholaminergic polymorphic ventricular tachycardia is a primary arrhythmogenic syndrome with genetic features most commonly seen in adolescents, with syncope and sudden death following exercise or agitation as the main clinical manifestations. The mechanism of its occurrence is related to the aberrant release of Ca^2+^ from cardiomyocytes caused by abnormal RyR2 channels or CASQ2 proteins under conditions of sympathetic excitation, thus inducing a delayed posterior exertional pole, manifested by sympathetic excitation inducing adrenaline secretion, resulting in bidirectional or polymorphic ventricular tachycardia. The mortality rate of the disease is high, but patients usually do not have organic heart disease, the clinical manifestations may not be obvious, and no significant abnormal changes in the QT interval are often observed on electrocardiography. Therefore, the disease is often easily missed and misdiagnosed. A number of genetic mutations have been linked to the development of this disease, and the mechanisms are different. In this paper, we would like to summarize the possible genes related to catecholaminergic polymorphic ventricular tachycardia in order to review the genetic tests currently performed, and to further promote the development of genetic testing techniques and deepen the research on the molecular level of this disease.

Catecholaminergic polymorphic ventricular tachycardia is a primary arrhythmia syndrome with genetic features. It is most common in children, and averagely, the first onset usually occurs at the age of 7–9 yr (Priori et al., 2002). The main clinical manifestation of CPVT is syncope after exercise or emotional excitement, or the progression to sudden cardiac death (Tester et al., 2019). Related studies show that the incidence of CPVT is about 1/10,000 (Leenhardt et al., 2012), the mechanism of its occurrence primarily involves abnormal calcium homeostasis in cardiomyocytes, usually caused by delayed posterior depolarization induced by abnormal RyR2 channels or CASQ2 proteins under conditions of sympathetic excitation, which proceeds to the development of bidirectional or polymorphic ventricular tachycardia (Priori et al., 2013). The lethality rate of CPVT is around 30–50% (Laitinen et al., 2001), and a related study shows that about 15% of sudden unexplained deaths with negative autopsy reports may have died from CPVT (Dharmawan et al., 2019). Patients with CPVT usually do not have organic heart disease and may have no obvious clinical symptoms and no obvious abnormal changes in the QT interval on ECG. Therefore, this disease is often easily missed and misdiagnosed. Several studies have suggested that CPVT has become an important cause of sudden cardiac death in children (van der Werf et al., 2010). Thus, gene screening plays a crucial role in assisting in the diagnosis of CPVT.

## Pathogenesis

There are two types of genes that have been found to be associated with the occurrence of CPVT: one is related to the gene mutations of cardiomyocyte ryanodine receptor (RyR2), which is CPVT1, accounts for about 50–60%; the other is related to the gene mutations of cardiomyocyte calcium collecting protein (CASQ2), which is CPVT2, accounts for about 3–5%. Hayashi et al. (Hayashi et al., 2009) have shown that the regulation of calcium ions (Ca^2+^) inside and outside of cardiomyocytes is precisely by the involvement of sodium-calcium exchangers, cell membrane voltage-gated calcium channels aslo called L-type calcium channels (LTCC), and RyR2 on the sarcoplasmic reticulum in conjunction with calcium collecting proteins in the sarcoplasmic reticulum lumen.

**FIGURE 1:**
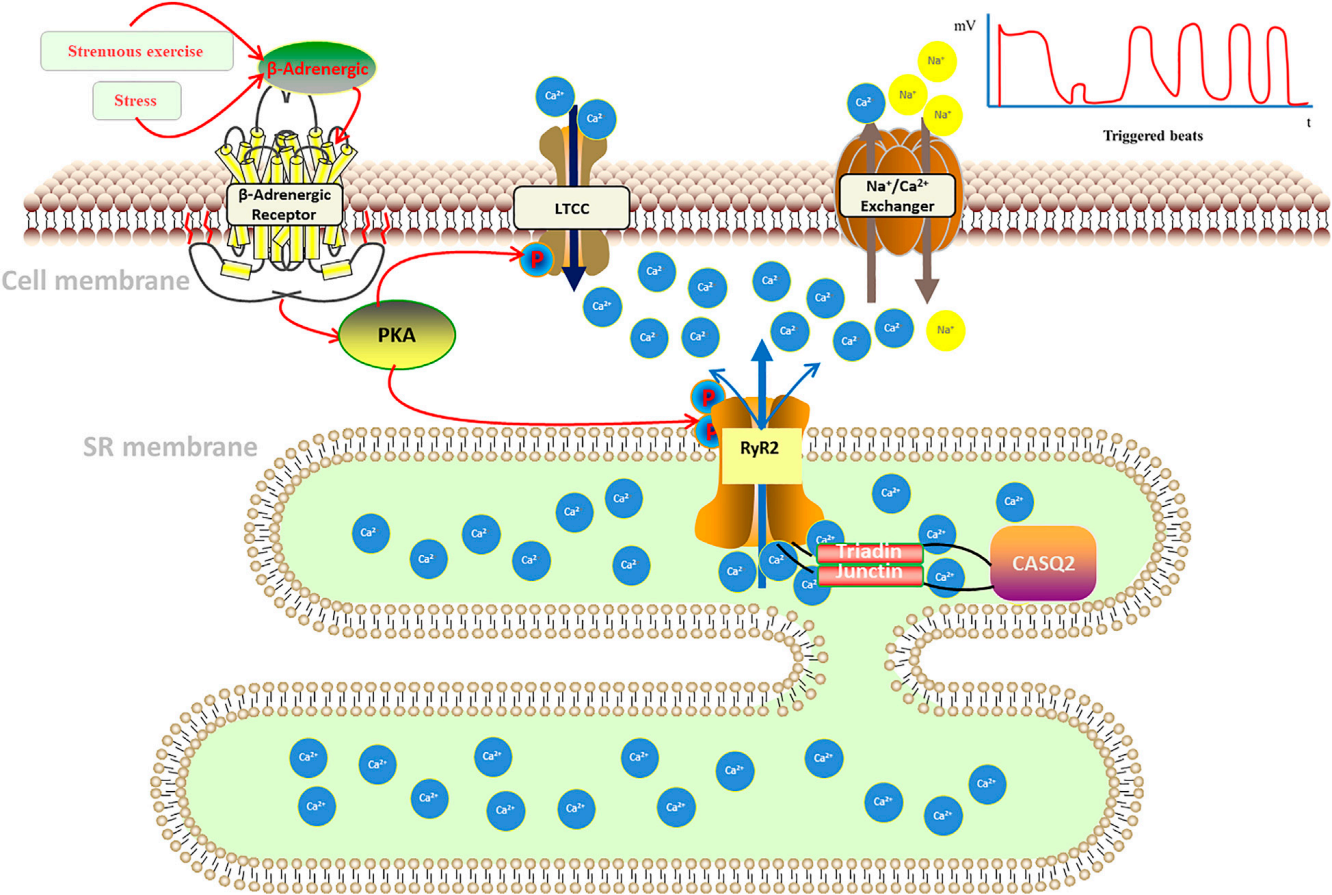
Pathogenesis of catecholaminergic polymorphic ventricular tachycardia.

Catecholamines activate GTP-binding proteins by activating β-adrenoceptors, and the protein can activate adenylate cyclase, increasing intracellular cyclophosphate adenosine concentrations and activating protein kinase A (PKA). Also, PKA will bind to RyR2 and activates it through the interaction of regulatory subunits with cyclic adenosine phosphate, which regulates the cardiomyocyte intracellular Ca^2+^ release (Bovo et al., 2017). Fabiato et al. (Fabiato and Fabiato, 1978) found that voltage-gated L-type calcium channels (LTCC) in cardiomyocyte membranes open in response to cardiomyocyte excitation, facilitating Ca^2+^ flow into cytoplasm, thus, increasing cardiomyocyte calcium load and sarcoplasmic reticulum (SR) calcium ingestion, then activating the ryanodine receptor (RyR) on the myoplasmic reticulum. This receptor is located on the intracellular sarcoplasmic reticulum and assists in the release of Ca^2+^ from the sarcoplasmic reticulum into the cytoplasm. We called the process calcium-induced calcium-release (CICR) (Figure 1) (Fabiato, 1985). On the other hand, calcium storage in the sarcoplasmic reticulum reaches a threshold, inducing spontaneous calcium release from the sarcoplasmic reticulum, a process named store-overload-induced calcium-release (SOICR) (Figure 1) (Jiang et al., 2004). When there is RyR2 mutation, the RyR2 calcium release channel in the sarcoplasmic reticulum becomes more sensitive to calcium in the sarcoplasmic reticulum lumen, making the channel open at lower calcium concentrations in the sarcoplasmic reticulum lumen, that will lower the calcium threshold at which results in store-overload-induced calcium-release. The both of mechanisms above will increase the concentration of calcium in intracellular plasma, which activates sodium-calcium exchangers in the myocardial cell membrane, resulting in an arrhythmogenic transient inward current that leads to cell membrane depolarization, also known as delayed afterdepolarization (DADS), which triggers pacing and the development of ventricular arrhythmias. And it is demonstrated by a dramatic increase in cytoplasmic Ca^2+^ concentration, which alters the conformation of troponin C in the myogenic fibers, causing displacement of the promyosin filaments, allowing the cross-bridges of the thick myofilaments to bind to the active sites of the thin myofilaments, resulting in myofilament gliding causing myocyte contraction.

In addition, calsequestrin (CASQ2), a calcium storage protein located in the lumen of the sarcoplasmic reticulum, also plays an important role in the regulation of Ca^2+^ in cardiomyocytes. CASQ2 protein is a calcium ion storage protein that regulates Ca^2+^ release from the sarcoplasmic reticulum by forming a complex with RyR2 (Mohamed et al., 2007). Normal CASQ2 protein polymerizes with RyR2 as the concentration of Ca^2+^ in the sarcoplasmic reticulum increases, and binds to RyR2 by forming complexes with triadin (TRDN) and junctin to reduce the opening of RyR2 channels, thereby limiting the transfer of Ca^2+^ from the sarcoplasmic reticulum to the cytoplasm (Knollmann et al., 2006; Song et al., 2007; Manno et al., 2017). When the CASQ2 protein is abnormal, Ca^2+^ in the sarcoplasmic reticulum spontaneously leaks into the cytoplasm, which also causes a delay in post-depolarization of cardiomyocytes, resulting in arrhythmias (Figure 1).

The rest of the genes associated with CPVT usually affect normal calcium channels by acting on the RYR2 and CQSA2 proteins, which will change the structure of the normal proteins that control the transfer of Ca^2+^ in cardiomyocytes, resulting in abnormal Ca^2+^ concentrations causing arrhythmias.

Catecholamines released from stress or exercise activate β-adrenergic receptors, causing a series of cellular chain reactions that lead to uncontrolled pathological calcium release from the sarcoplasmic reticulum: *1*) calcium-induced calcium-release (CICR): when an action potential occurs in cardiomyocytes, the cell membrane depolarizes, opening L-type calcium channels (LTCC), allowing calcium to enter the cytoplasm, where intracellular calcium ions bind to the sarcoplasmic reticulum and stimulate the opening of RyR2 calcium release related channels on the sarcoplasmic. *2*) store-overload-induced calcium-release (SOICR): calcium storage in the sarcoplasmic reticulum reaches a threshold, inducing spontaneous calcium release from the sarcoplasmic reticulum. When there is RyR2 mutation, it will lower the calcium threshold at which results in store-overload-induced calcium-release. Both mechanisms lead to an increase in intracellular calcium concentration, which activates the sodium-calcium exchanger in the cytosol, generating an arrhythmogenic inward ion flow that delays membrane depolarization, triggering ventricular contraction, and inducing ventricular tachycardia (Kim et al., 2020; Wleklinski et al., 2020).

## Diagnostic Criteria

The American Heart Rhythm Society (HRS), the European Heart Rhythm Association (EHRA), and the Asia Pacific Heart Rhythm Society (APHRS) jointly developed a consensus on the diagnosis and treatment of hereditary primary arrhythmia syndrome (HPS) in 2013 (Priori et al., 2013). The consensus considers CPVT to be diagnosed when one of the following conditions is met: *1*) age <40 yr, normal cardiac structure and resting ECG, not otherwise explained by exercise or catecholamine-induced bidirectional ventricular tachycardia or polymorphic ventricular precontractions (premature ventricular contractions), ventricular tachycardia; *2*) patients carrying pathogenic mutations (prior or family members); *3*) family members with normal cardiac structure in CPVT preexisting patients members, who induce premature or bidirectional/polymorphic ventricular tachycardia after exercise. It has also been shown that exercise ECG and Holter monitoring play a prominent role in the diagnosis of CPVT.

## Related Genes

RyR2 mutations are most common in CPVT-related mutations. Yuchi et al. (Yuchi and Van Petegem, 2016) showed that RyR2, which is based on a homotetramer located on the sarcoplasmic reticulum with Ca^2+^ channels formed in its midst to transport Ca^2+^, plays a key role in maintaining calcium homeostasis in cardiomyocytes. The vast majority of RyR2 mutations result in increased RyR2 channel function, promoting greater Ca^2+^ translocation from the myoplasmic reticulum of cardiomyocytes into the cytoplasm. The gene encoding RyR2 has a total of 105 exons, and by summarizing RyR2-related mutation sites, Medeiros-Domingo et al. (Medeiros-Domingo et al., 2009) found that more than 90% of the mutations occur in three specific regions of the RyR2 protein amino acid linkage: the N-terminal, C-terminal transmembrane region and the FKBP12.6 (calcium channel stabilization protein 2) binding region located in the middle segment. Wehrens et al. (Wehrens et al., 2003) showed that the R4497C mutation in the RyR2 gene reduces the affinity of FKBP12.6 for RyR2, thereby triggering CPVT. Xu et al. (Xu et al., 2010) found that the R2474S site mutation in RyR2 causes spontaneous Ca^2+^ leakage causing arrhythmias. Mutations in the D3638A locus of RyR2 cause alterations in the spatial structure of post-transcriptional RyR2, resulting in Ca^2+^ leakage (Acimovic et al., 2018). In addition to the above-mentioned related sites, several studies have shown that mutations at multiple sites on the RyR2 gene can lead to CPVT in different altered ways, including G203C, V4653F, S2246L, N4104K, and P2328S (Priori and Chen, 2011; Venetucci et al., 2012; Leinonen et al., 2018).

Calsequestrin 2 (CASQ2) is a Ca^2+^ storage protein and its associated gene encodes cardiac troponin, which is often mutated in an autosomal recessive manner (Lahat et al., 2001). CASQ2, through its association with RyR2 and other proteins: connexin (Junctin) and triadin (triadin, TRDN) and other interactions to control the storage and release of Ca^2+^ in the sarcoplasmic reticulum (Gaburjakova et al., 2013). When the relevant gene is mutated, the spatial structure of the CASQ2 protein is altered, which affects the calcium homeostasis in the cytoplasm and leads to arrhythmias (Katz et al., 2009). Mutations in CASQ2 can be divided into pure mutations and complex heterozygous mutations. Viatchenko-Karpinski S et al. (Viatchenko-Karpinski et al., 2004) found that the fragment mutations on CASQ2, D307H can influence the Ca^2+^ reserve capacity in the intracellular sarcoplasmic reticulum, which further interferes with Ca^2+^-induced calcium release. Roux-Buisson N et al. (Roux-Buisson et al., 2011) found that synonymous mutations caused by mutations in the intron c.939+5G>C point on CASQ2-related genes lead to structural changes in the associated proteins, which will cause CVPT. Terentyev D et al. (Terentyev et al., 2008) reported that the R33Q mutation affects the binding of RyR2 to CASQ2 and interferes with intracellular Ca^2+^ transport causing cardiac arrhythmias. In contrast to pure mutations, heterozygous mutations on CASQ2-related genes are more complex. In addition, Lys180ARG was found to be a heterozygous missense variant of the CASQ2 gene that affects highly conserved residues and was not found in unaffected family members (Gray et al., 2016). di Barletta MR et al. (di Barletta et al., 2006) were the first to report a complex heterozygosity from CASQ2-G11^2+^5X/L167H. This mutation caused abnormal Ca^2+^ regulation in the sarcoplasmic reticulum to trigger arrhythmias. One case of CPVT caused by a compound heterozygous mutation of CASQ2-c.199C > T (p.Gln67*)/c.53^2+^1G> A has also been reported (Josephs et al., 2017). More researches are still required to further recognize the mutational pattern of CASQ2.

Mutations in the TRDN gene have also been implicated in the development of CPVT (Landstrom et al., 2017), which encodes a tripeptide protein, a transmembrane protein located in the intracellular sarcoplasmic reticulum, that interacts with CASQ2, RyR2, and other proteins and is critical for the regulation of Ca^2+^ stores and release. Mutations in the TRDN gene result in a significant decrease in protein expression and cause Ca^2+^ overload in the sarcoplasmic reticulum, which contributes to spontaneous Ca^2+^ release leading to the development of CVPT. Rooryck C et al. (Rooryck et al., 2015) clearly associated heterozygous mutations on the TRDN gene (c.613C > T/p.Gln205 * and c.22 + 29 A> G) with the development of CPVT by studying a group of CPVT families. O′Callaghan BM et al. (O'Callaghan et al., 2018) also reported a case of CPVT in which genetic testing revealed a deletion of exon 3 of the TRDN gene.

The CaM protein is a calmodulin, and its related genes can be divided into three categories: CALM1, CALM2 and CALM3, which are responsible for encoding the same CaM. CaM protein abnormality affects the release of Ca^2+^ from RyR2, which in turn causes abnormal Ca^2+^ concentration in cardiomyocytes and leads to arrhythmias. Nyegaard M et al. (Nyegaard et al., 2012) demonstrated that mutations in CALM1 and CALM2 genes are all associated with the development of CPVT. Some authors have also detected a mutation in the CALM3-A103V locus in one patient with CPVT and suggested that the mutation may alter the binding of CaM protein to Ca^2+^, thus affecting the intracellular Ca^2+^ ion concentration (Gomez-Hurtado et al., 2016).

The protein encoded by the trans-2,3-enoyl-CoA reductase-like protein (TECRL) gene contains 363 amino acids, with a total of 12 exons, is expressed in the myogenic reticulum of cardiac and skeletal muscle and is mainly involved in the regulation of the metabolism of lipid components (Xie et al., 2019). Through multiple family studies, several studies have shown that pure mutations at multiple loci on the TECRL gene can cause arrhythmias due to abnormal Ca^2+^ concentrations in cardiomyocytes (Devalla et al., 2016; Jalloul and Refaat, 2020). Xie et al. (Xie et al., 2019) demonstrated that the R196Q mutation in the TECRL gene can cause CPVT. While Devalla et al. (Devalla et al., 2016) compared the pure mutation and heterozygous mutation of R196Q in TECRL gene with the wild type of TECRL gene, discovering that both the pure mutation and heterozygous mutation can cause a decrease in Ca^2+^ stores in the sarcoplasmic reticulum of cardiomyocytes and an increase in diastolic cytoplasmic Ca^2+^ concentration in cardiomyocytes, which leaded to CVPT. But the exact mechanism is still unclear.

Kir2.1, encoded by KCNJ2 gene, is a subunit of the inward-rectifier potassium channels Ik1 in cardiomyocyte. It has reported that the structural and functional abnormalities of the inwardly rectifying potassium channel Ik1 play an important role in calcium-dependent and triggered arrhythmias (Pogwizd et al., 2001). Amanda et al. found a KCNJ2 mutation (p.V227F) in a CPVT patient. And in a cytology experiment, by using forskolin and 3-isobutyl-1-methylxanthine to imitate catecholamine action to increase PKA activity, they identified that the mutation made no functional defects on motionless Kir2.1 channels, while it only resulted in the loss-of-function regarding the cAMP-dependent protein kinase A (PKA)-dependent phosphorylation of Kir2.1 channel proteins. It reduced the inward potassium current, unstabilizing the membrane current and causing ventricular tachycardia. Thus, it indicated that there is some relevance between the KCNJ2 mutation with CPVT (Vega et al., 2009). In addition, several studies have also reported that KCNJ2 mutations are associated with catecholaminergic polymorphic ventricular tachycardia (Kawamura et al., 2013).

Ank2 gene encodes ankyrin-B protein, and ankyrin-B is the important part of Na^+^/Ca^2+^ exchanger, Na^+^/K^+^ ATPase and inositol trisphosphate (InsP3) receptor which locates mainly in transverse-tubule/sarcoplasmic reticulum sites of cardiomyocytes. Loss-of-effect in ankyrin-B leads to the abnormal expression and function of Na^+^/Ca^2+^ exchanger, Na^+^/K^+^ ATPase and InsP3 receptor, which affecting the conduction and rhythm of cardiomyocytes and the function of sinus and atrioventricular nodes. It has reported that patients with ankyrin-B mutations show varying types of cardiac arrhythmia, which including catecholaminergic polymorphic ventricular tachycardia (Mohler et al., 2004).

PKP2 gene encodes plakophilin-2 protein. Absence of PKP2 gene obviously reduces the transcriptional expression of genes including RYR2、TRDN and Ank2 et al. which are necessary for intracellular calcium tansfer, and also reduces the level of calsequestrin-2. It would influence the intracellular calcium stabilization, leading to catecholaminergic polymorphic ventricular tachycardia (Tester et al., 2019). In a study, Delmar et al. (Cerrone et al., 2017) demonstrated that the PKP2-knockout mice model exhibited the clinical manifestation of CPVT.

Junctin protein is encoded by JUN gene which is also called aspartyl-beta-hydroxylase (ASPH) gene (Wleklinski et al., 2020). Larry R. et al. found there were distinct homologies between junctin and triadin、ASPH, indicating that they all are the members of endoplasmic reticulum proteins (Jones et al., 1995). Junctin is located in the junctional part of the sarcoplasmic reticulum in cardiomyocyte. Resembling triadin, junctin, as the scaffold part of calsequestrin, participates in the accumulation of calcium in the sarcoplasmic reticulum. On the other hand, calsequestrin is grappled to RyR2 by triadin and junctin, regulating the release of calcium (Kobayashi and Jones, 1999). CPVT as a familial arrhythmia, is generated by the abnormal release of calcium in the sarcoplasmic reticulum, and is associated significantly with RYR2 and CASQ2 gene. Moreover, junctin influences calcium channel by functioning on CASQ2 protein, causing aberrant calcium release which results in CPVT. Thus, the JUN mutation is linked to CPVT (Lee et al., 2012).

The gene SCN5A mainly encodes sodium channel NaV1.5 in cardiac. And the NaV1.5 is the principal channel for maintaining the cardiac sodium current (INa), which ensures the rapid upstroke of the cardiac action potential. Therefore, it takes a crucial impact in cardiac electrophysiology (Veerman et al., 2015). Studies found a missense mutation (p.I141V) in a highly conserved region of the SCN5A gene, causing the lose-of-function in the Nav1.5 sodium channel protein, which leads to the abnormal sodium current and lowers the excitability threshold of cardiomyocyte. It results in exercise-induced polymorphic ventricular arrhythmias (Swan et al., 2014). Mohamed et al. suggested that the SCN5A mutations may result in exercise-induced polymorphic ventricular tachycardia resembling CPVT (Amarouch et al., 2014). However, the correlation between SCN5A mutations and CPVT yet needs to be further confirmed.

In addition to the above, it also has been confirmed that the KCNQ1 and NKYRIN-B gene mutations are related to the occurrence of CPVT, but the relevant mechanisms are still unclear (Velcea et al., 2017). And some studies have found mutations in KCNE1、KCNE2 and KCNH2 gene in the patients with CPVT, but it is still unclear for the correlation between those genes and CPVT (Table 1).

**TABLE 1:** Relevant genes.

	Gene	Encodes proteins	Mechanism
Explicitly relevant	RyR2	Ryanodine-recepter-relativeCa^2+^-release-channels	Influences RyR2 channel function, regulating the cardiomyocyte intracellular Ca^2+^ release
	CASQ2	Calsequestrin2 protein	Reduces the opening of RyR2 channels, limiting the transfer of Ca^2+^ from the sarcoplasmic reticulum to the cytoplasm
	TRDN	triadin protein	Interacts with CASQ2, RyR2, and other proteins, regulating the Ca^2+^ stores and release
	CALM1, CALM2, CALM3	Calmodulin CaM protein	Affects the release of Ca^2+^ from RyR2, which in turn causes abnormal Ca^2+^ concentration in cardiomyocytes
Closely relevant	TECRL	Trans-2,3-enoyl-CoA reductase-like protein	Expresses in the myogenic reticulum of cardiac, mainly involved in the regulation of the metabolism of lipid components
	KCNJ2	Inward-rectifier potassium channels Kir2.1	Reduces the inward potassium current, influencing the membrane current and stabilizes the Ca^2+^ in cardiomyocyte
	AnK2	Ankyrin-B protein	Affects the expression and function of Na^+^/Ca^2+^ exchanger, Na^+^/K^+^ ATPase and InsP3 receptor, which changing the conduction and rhythm of cardiomyocytes and the function of sinus and atrioventricular nodes
	PkP2	Plakophilin-2 protein	Affects expression of critical genes including RYR2、TRDN and Ank2 et al. which are necessary for intracellular calcium transfer
	JUN	Junctin protein	Participates in the accumulation of calcium in the sarcoplasmic reticulum as the scaffold part of calsequestrin
	SCN5A	Sodium channel NaV1.5	Affects NaV1.5 sodium channel protein activity, maintaining the excitability threshold of cardiomyocyte
Potentially relevant	KCNQ1, NKYRIN-B, KCNE1, KCNE2, KCNH2	Unclear	Unclear

## Treatments

CPVT is prone to dangerous complications such as ventricular fibrillation, syncope, and even sudden cardiac death, and must be intervened and treated as soon as possible. The current consensus on the treatment of CPVT includes drug therapy, surgery, and gene therapy, of which gene targeted therapy is under research as a hot new treatment method. As CPVT occurs more often after exercise or emotional stress, it is also important to avoid strenuous exercise, emotional stress, and arrhythmia-inducing drugs, etc. Patients with CPVT usually do not have organic heart disease, and the manifestations of CPVT are diverse, which makes it liable to misdiagnose or never diagnose, so regular follow-up and individualized treatment according to risk stratification are needed.

### Medical Therapy

#### β-adrenergic Receptor Inhibitors

On account of CPVT is triggered by β-adrenergic stimulation, β-blockers are recommended as first-line and basic medications according to the 2013 consensus on arrhythmia practice (Priori et al., 2013). Clinical studies by Hayashi (Hayashi et al., 2009) and Leren (Leren et al., 2016) have confirmed that non-selective β-blockers (e.g., nadolol, propranolol) are therapeutically superior to selective beta-blockers, and nadolol is the most effective. But the mechanism why it is more effective in still unclear.

#### Flecainide and Propafenone

As the class Ic antiarrhythmic drugs, flecainide and propafenone can reduce the occurrence of arrhythmia in some patients with CPVT (Bannister et al., 2015). In a clinical study, Roston et al. found that the combination of β-blockers and foscarnet significantly reduced the occurrence of malignant arrhythmia without increasing the occurrence of side effects (Roston et al., 2015). While at present, the clinical application of foscarnet and propafenone is not widespread, and the specific mechanism is still unclear, which needs to be confirmed by further experimental studies.

### Surgery

#### Implantable Cardiac Defibrillator (ICD)

The 2013 guidelines recommended that ICDs should be implanted in patients with CPVT who have a history of syncope or cardiac arrest after receiving regular and effective drug therapy (Priori et al., 2013). But recent associated studies have reported that ICDs have abnormal discharges and other adverse events. A meta-analysis including 1,429 patients with CPVT by Roston et al. showed that, 40% patients with ICDs had at least one shock due to abnormal ICD discharges; 20% had electrical storms, which means three or more episodes of persistent ventricular tachycardia, ventricular fibrillation, or shock triggered by the ICD within 24 h; and four of the seven patients with CPVT who died after ICD implantation died from electrical storms (Roston et al., 2018). A clinical study by van der Werf et al. (van der Werf et al., 2019) showed that some patients with CPVT did not benefit from ICDs, which suggests ICD implantation did not prevent the development of malignant arrhythmias. These data and studies indicate that the treatment of CPVT with ICDs is controversial and it must be evaluated by physicians with extensive knowledge and experience in this area before it is applied.

#### Left Cardiac Sympathetic Denervation (LCSD)

LCSD can more completely prevent the development of CPVT by removing sympathetic nerves, reducing catecholamine secretion, and decreasing the effect of catecholamines on cardiomyocytes (Sumitomo, 2016). Recent guidelines have suggested that LCSD may be an alternative treatment option for patients with CPVT who remain symptomatic despite optimal medical therapy or who have frequent abnormal discharges after ICD implantation. However, LCSD requires a high level of competence of the surgeon and the medical equipment of the hospital, so the implementation is still difficult.

### Gene Targeted Therapy

As the increasing research on gene therapy, gene targeted therapy will have the potential to replace drug or surgical treatment. Several experimental studies have successfully targeted to treat hereditary arrhythmias in CPVT mouse models by adeno-associated virus (AAV) vectors. Denegri et al. (Denegri et al., 2012; Denegri et al., 2014) (2012, 2014), Kurtzwald-Josefson et al. (Kurtzwald-Josefson et al., 2017) (2017), Cacheux et al. (Cacheux et al., 2020) (2020) transfected CASQ2 gene to CASQ2 knockout mice with adeno-associated virus (AAV), and the experimental results suggested that the expression levels of CASQ2, Triadin and Junctin proteins on the myoplasmic reticulum of transfected mouse cardiom yocytes were significantly improved, which ensured normal calcium release and effectively prevented the occurrence of CVPT. Bongianino et al. (Bongianino et al., 2017) (2017), Liu et al. (Liu et al., 2018) (2018), Pan et al. (Pan et al., 2018) (2018) reduced the incidence of isoprenoid-induced ventricular arrhythmias by targeting to silence the RyR2 mutant genes to inhibit abnormal calcium release due to RyR2 mutations. The above studies demonstrate the ability of gene targeting to treat CPVT, and as more CPVT-related gene targeting is investigated and confirmed, hereditary arrhythmia disease is expected to be cured.

## Discussion

Catecholaminergic polymorphic ventricular tachycardia is a primary arrhythmia syndrome with genetic features most commonly seen in adolescents, with syncope and sudden death following exercise or agitation as the main clinical manifestations. CPVT has a high mortality rate, but patients usually do not have organic heart disease, the clinical presentation may not be obvious, and no significant abnormal changes in the QT interval are often observed on ECG. Therefore, this disease is often prone to be missed and misdiagnosed. At the same time, CPVT has obvious family heritability, which suggests that genetic testing has an important role in assisting the diagnosis of CPVT. Now genetic testing is available to determine the presence of relevant disease-causing mutations to further clarify the diagnosis of CPVT. Genetic testing is necessary to enable early diagnosis and treatment of patients with suspected CPVT. It is worth mentioning that early diagnosis of CPVT can help to carry out correct treatment early, which can substantially improve the quality of survival and prognosis of children with CPVT. On the other hand, most children with CPVT will improve effectively with beta-blocker therapy, but some children still have poor outcomes. Several studies have been conducted to apply gene targeted therapy for hereditary arrhythmias in the mouse model of CPVT. With the advancement of genetic technology, we believe that more targeted therapies for CPVT-related genes can be studied and confirmed, and hereditary arrhythmia diseases are expected to be cured.

However, about one-third of patients with CPVT still do not have a clear genetic diagnosis, and the mechanism of CPVT which caused by several gene mutations is still unclear yet. We need to continue to use bioinformatics statistical analysis and high-throughput gene sequencing technology to find more relevant genes, to clarify the specific genetic mechanism and the pathogenesis of the relevant genes, through subsequent further functional experiments, such as, gene overexpression experiments, RNA interference experiments in cell-level and transgenic experiments, gene-knockout experiments in animal-level. Therefore, we can make a clear genetic diagnosis and carry out early treatment.
